# Integrative genomics approach identifies molecular features associated with early-stage ovarian carcinoma histotypes

**DOI:** 10.1038/s41598-020-64794-8

**Published:** 2020-05-14

**Authors:** Hanna Engqvist, Toshima Z. Parris, Jana Biermann, Elisabeth Werner Rönnerman, Peter Larsson, Karin Sundfeldt, Anikó Kovács, Per Karlsson, Khalil Helou

**Affiliations:** 1 Department of Oncology, Institute of Clinical Sciences, Sahlgrenska Cancer Center, Sahlgrenska Academy at University of Gothenburg, Gothenburg, Sweden; 2 Sahlgrenska University Hospital, Department of Clinical Pathology, Gothenburg, Sweden; 3 Department of Obstetrics and Gynecology, Institute of Clinical Sciences, Sahlgrenska Cancer Center, Sahlgrenska Academy at University of Gothenburg, Gothenburg, Sweden

**Keywords:** Ovarian cancer, Cancer genomics, Methylation analysis

## Abstract

Ovarian cancer comprises multiple subtypes (clear-cell (CCC), endometrioid (EC), high-grade serous (HGSC), low-grade serous (LGSC), and mucinous carcinomas (MC)) with differing molecular and clinical behavior. However, robust histotype-specific biomarkers for clinical use have yet to be identified. Here, we utilized a multi-omics approach to identify novel histotype-specific genetic markers associated with ovarian carcinoma histotypes (CCC, EC, HGSC, and MC) using DNA methylation, DNA copy number alteration and RNA sequencing data for 96 primary invasive early-stage (stage I and II) ovarian carcinomas. More specifically, the DNA methylation analysis revealed hypermethylation for CCC in comparison with the other histotypes. Moreover, copy number imbalances and novel chromothripsis-like rearrangements (n = 64) were identified in ovarian carcinoma, with the highest number of chromothripsis-like patterns in HGSC. For the 1000 most variable transcripts, underexpression was most prominent for all histotypes in comparison with normal ovarian samples. Overall, the integrative approach identified 46 putative oncogenes (overexpressed, hypomethylated and DNA gain) and three putative tumor suppressor genes (underexpressed, hypermethylated and DNA loss) when comparing the different histotypes. In conclusion, the current study provides novel insights into molecular features associated with early-stage ovarian carcinoma that may improve patient stratification and subclassification of the histotypes.

## Introduction

In recent years, it has been shown that ovarian carcinoma comprises five main histotypes, namely clear-cell (CCC), endometrioid (EC), high-grade serous (HGSC), low-grade serous (LGSC) and mucinous carcinomas (MC). Multiple studies have demonstrated that the histotypes differ in terms of *e.g*. origin, risk factors, prognosis, and molecular and clinical behavior^1–3^. Furthermore, the ovarian carcinoma histotypes exhibit mutation-specific profiles, *e.g*. HGSC is characterized by recurrent *TP53* mutations, whereas EC and CCC often comprise mutations in the *ARID1A* and *PIK3CA* genes^4,5^. Comprehensive characterization of epigenetic and copy number alterations (CNAs) in the different histotypes are however less documented^6–8^. Today, a wide range of multi-omics data, *e.g*. genome-, transcriptome- and epigenome-wide analyses, are available that permit the characterization of molecular events underlying the development and progression of cancer. Different molecular mechanisms may influence gene expression during cancer initiation and progression, thereby contributing to altered expression of genes important in tumorigenesis. More specifically, gene expression is affected by *e.g*. germline and somatic factors, CNAs and epigenetic events, such as DNA methylation changes^9,10^. Therefore, integrated multi-omics analyses may potentially allow the identification of more robust biomarkers for individualized clinical decision-making^11^. However, few integrative molecular studies have to date been performed for the different ovarian carcinoma histotypes that could give greater insight into molecular events characterizing these disease-states.

The Cancer Genome Atlas (TCGA) ovarian carcinoma cohort has provided comprehensive genetic (exome sequencing, mRNA, microRNA), epigenetic (promoter methylation) and DNA CNA data, but is currently limited to only HGSC patients^4^. A recent report evaluated DNA methylation patterns and CNA data for the different histotypes^7^. However, the CNA data (180 K-feature array comparative genomic hybridization (aCGH)) was only provided for a subgroup of the samples (47/162 samples) profiled for modulations in DNA methylation patterns. Although the DNA methylation data was also compared with NanoString gene expression data (n = 518 genes), the analysis was limited to HGSC patients (61/162 samples). Hence, truly integrated omics-wide analyses containing the same ovarian carcinoma cohort have yet to be performed.

Here, we performed a comprehensive genome- and transcriptome-wide analysis integrating DNA methylation, CNA and RNA sequencing (RNA-seq) data for 96 primary invasive early-stage (stage I and II) ovarian carcinoma samples characterized as CCC, EC, HGSC and MC. Omics-wide integrated analyses have not previously been performed for cohorts containing samples from early-stage patients. Based on the assumption that the genetic profiles of early-stage tumors are generally less complex compared to the later stages, we chose to only include early-stage ovarian carcinoma to enable the classification of early events in ovarian carcinoma tumorigenesis. This may permit the identification of specific genomic alterations related to ovarian carcinoma. Large-scale identification of molecular features in ovarian carcinomas may provide important insight into key molecular characteristics differing between the histotypes, enabling improved histotype classification and may in the future contribute to improved treatment strategies for specific histotypes. Here, we provide an extensive overview of the genome, methylome, and transcriptome for early-stage ovarian carcinomas, thereby identifying putative genetic markers for ovarian carcinoma, such as oncogenes and tumor suppressor genes.

## Results

### Differential DNA methylation analysis revealed hypermethylation in CCC

**Figure 1:**
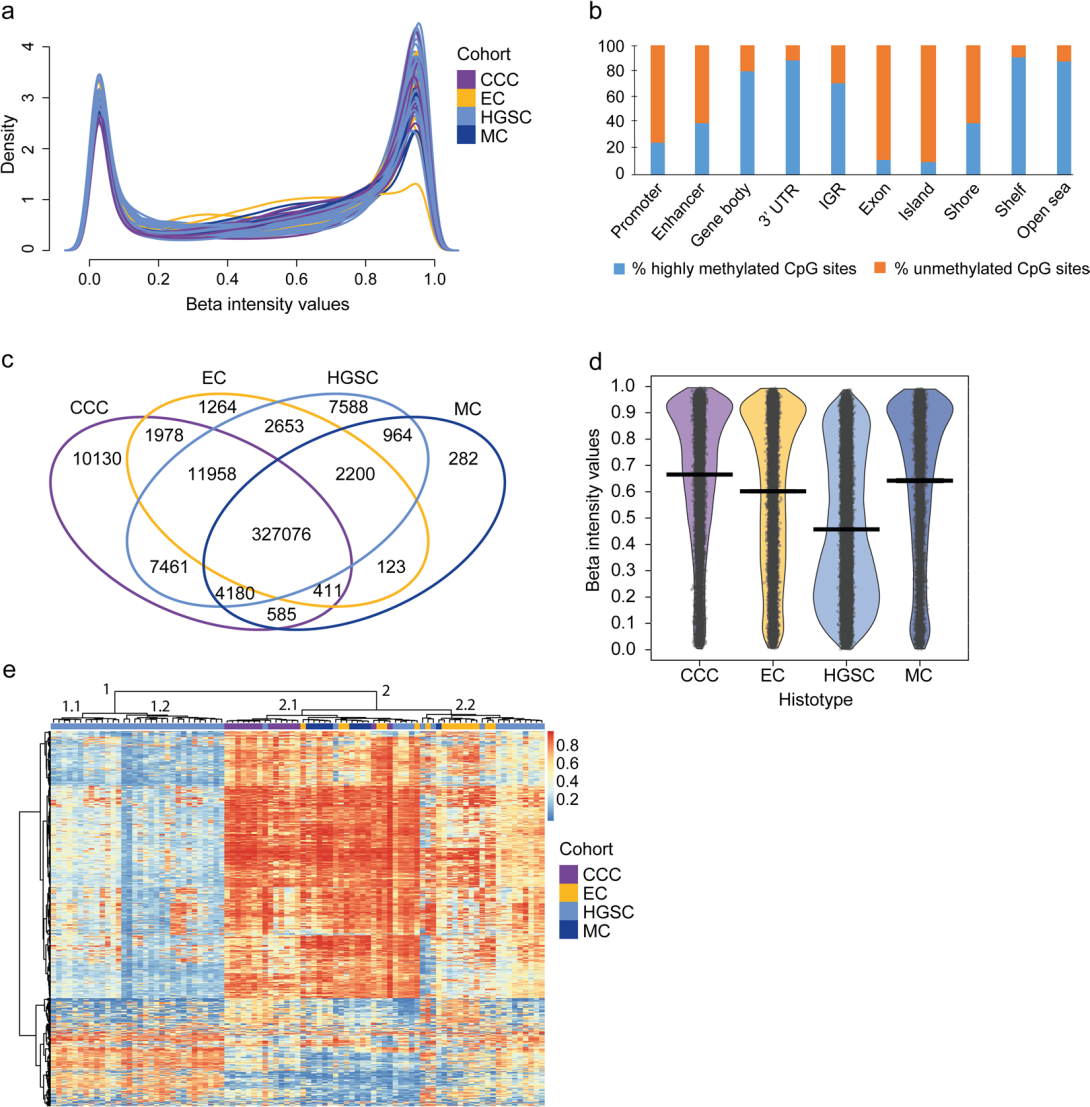
DNA methylation pattern in ovarian carcinoma. DNA methylation density plot (**a**) showing the beta value distribution of individual probes (n = 679,259) after batch correction colored by the ovarian carcinoma histotypes (clear-cell (CCC), endometrioid (EC), high-grade serous (HGSC) and mucinous (MC) ovarian carcinomas). Overall, more highly methylated probes were identified in the cohort. The x-axis denotes beta intensity values and the y-axis density. Column chart (**b**) showing an overview of the relative distribution of methylation patterns across genomic regions. Unmethylated CpG sites were more common in the promoter, enhancer, exon, CpG island and shore, while highly methylated CpG sites were prevalent in the gene body, 3′ UTR, IGR, shelve and open sea. Venn diagram (**c**) showing unique and overlapping differentially methylated probes (DMPs) between histotypes (*P* value<0.05). The highest number of unique DMPs was identified in CCCs, whereas 300,406 DMPS were not differentially methylated in any histotype comparison. RDI (Raw data, Descriptive, Inference statistics) plot (**d**) showing the difference in methylation patterns between histotypes for the 1,000 most variable probes. Black open circles distributed horizontally represent raw data probes and the surrounding colored beans depict smoothed densities. A large proportion of probes were methylated in CCC (mean depicted by the black vertical center bar), whereas probes were predominantly unmethylated in HGSCs. Significant differences in DNA methylation patterns were found for all histotypes (Wilcoxon test). CCC vs MC comparison had a *P* value<0.01 and the remaining comparisons had *P* values<0.0001. Heatmap (**e**) of DNA methylation beta values for the 1,000 most variable probes across the cohort. Red color represents highly methylated probes and blue color unmethylated probes. Ward’s method was used for the hierarchical clustering of histotypes (colored bar at the top of the heatmap) and Canberra distance measure was used to calculate the dissimilarity measure for the heatmap. On the left side of the heatmap the 1,000 CpG sites are clustered into two main clusters.

A comprehensive DNA methylation analysis was performed using 91 early-stage ovarian carcinoma samples of various histotypes (CCC, EC, HGSC, MC). After batch correction of biological and technical parameters, histotype and survival were shown to still have significant effects on DNA methylation (Supplementary Fig. 1). In general, DNA methylation (beta values > 0.8) was prevalent in ovarian carcinoma (Fig. 1a). On one hand, unmethylated CpG sites were more prevalent in specific genomic regions including promoter, enhancer and exon, as well as in regions denoted as CpG island and shore. On the other hand, highly methylated CpG sites were more frequently found in the gene body, 3′ untranslated regions (3′ UTR) and intergenic regions (IGR), as well as in CpG shelves and open sea (Fig. 1b). Limma was then used to identify unique and overlapping differentially methylated probes between histotype groups, revealing 10,130 unique probes for CCC, 1,264 for EC, 7,588 for HGSC and 282 for MC (Benjamini-Hochberg adjusted *P* value<0.05; Fig. 1c, Supplementary Table 1). Less than half of the EPIC probes (n = 300,406) were not differentially methylated.

**Figure 2:**
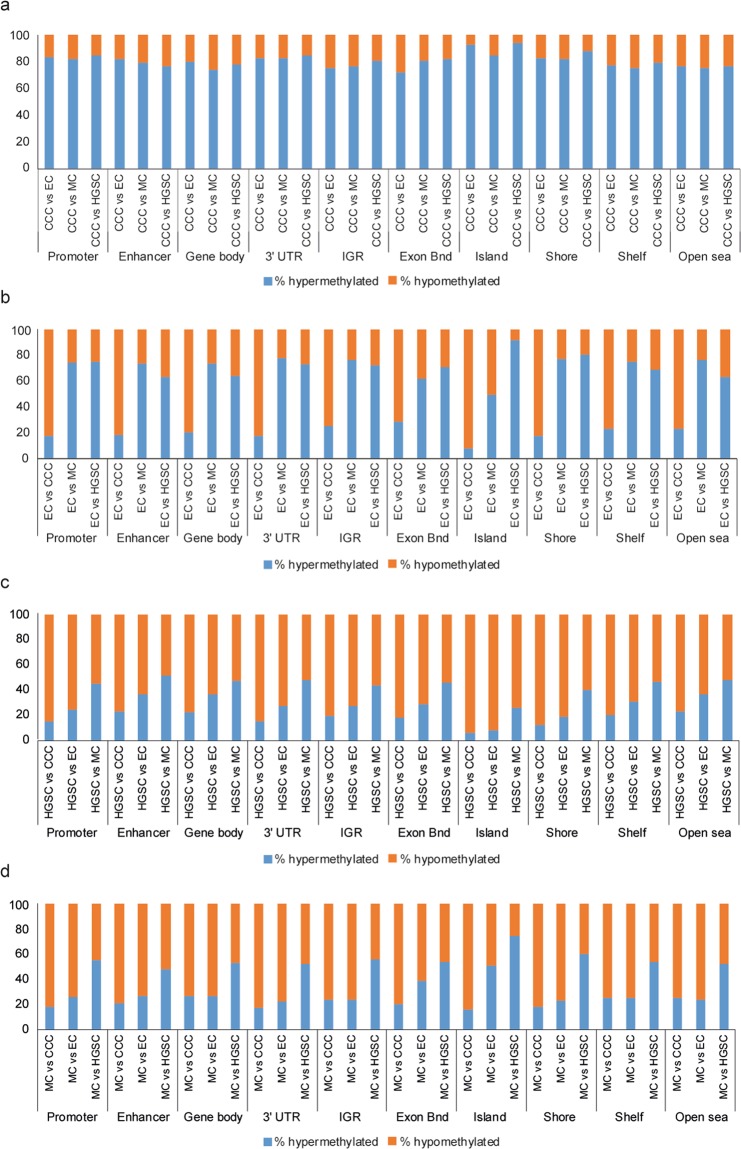
Relative distribution of DNA methylation patterns. Column charts showing the relative distribution of methylation patterns across genomic regions and regions surrounding CpG islands in CCC tumors compared with EC, MC and HGSC, respectively (**a**), EC as case (**b**), HGSC as case (**c**) and MC as case (**d**). Beta values greater than or equal to 0.2 denotes hypermethylated differentially methylated probes (DMPs) and beta values less than or equal to -0.2 denotes hypomethylated DMPs (Benjamini-Hochberg adjusted *P* value<0.05). The Promoter region refers to 200bp-1500bp upstream of transcriptional start sites, 1^st^ exon and 5′ UTR.

Histotype-specific methylation patterns were demonstrated for the 1,000 most variable probes across the cohort, with the highest mean distribution of methylated probes found in the CCC patient group, followed by MC and EC patients, while HGSCs showed the lowest mean distribution of methylated probes (Fig. 1d). It is also evident that HGSCs contained a higher number of unmethylated probes in comparison with CCC, EC and MC. Hierarchical clustering of the 1,000 most variable probes stratified the cohort into two main clusters (clusters 1 and 2), wherein cluster 1 only included HGSC tumor samples (Fig. 1e). Clusters 1 and 2 were further stratified into two sub-clusters each (clusters 1.1, 1.2 and 2.1, 2.2). Both CCC and MC clustered in sub-cluster 2.1 except one MC sample in sub-cluster 2.2, whereas EC was found in both sub-clusters (clusters 2.1 and 2.2). The DNA methylation probes also clustered into two main clusters (top and bottom CpG clusters). For the top CpG cluster, the HGSC samples generally demonstrated unmethylated CpG sites, while CCC and MC demonstrated highly methylated CpG sites. Differential methylation analysis using the differentially methylated probe (DMP) function in ChAMP revealed 13,003 DMPs (10051 hyper-, 2,952 hypomethylated DMPs) between CCC and MC, 6,732 DMPs (1,282 hyper-, 5,450 hypomethylated DMPs) between EC and CCC, 2,248 DMPs (1,660 hyper-, 588 hypomethylated DMPs) between EC and MC, 23,313 DMPs (4,596 hyper-, 18,717 hypomethylated DMPs) between HGSC and CCC, 10,626 DMPs (3,414 hyper-, 7,212 hypomethylated DMPs) between HGSC and EC comparison, and 26,515 DMPs (12,352 hyper-, 14,163 hypomethylated DMPs) between HGSC and MC comparison. Interestingly, CCC was generally hypermethylated for all genomic regions and regions surrounding CpG islands in all histotype comparisons (Fig. 2). EC was more hypermethylated in comparison with MC and HGSC, whereas HGSC and MC were predominantly hypomethylated in all histotype comparisons.

### DNA copy number alteration analysis revealed complex copy number imbalances and chromothripsis-like rearrangements

**Figure 3:**
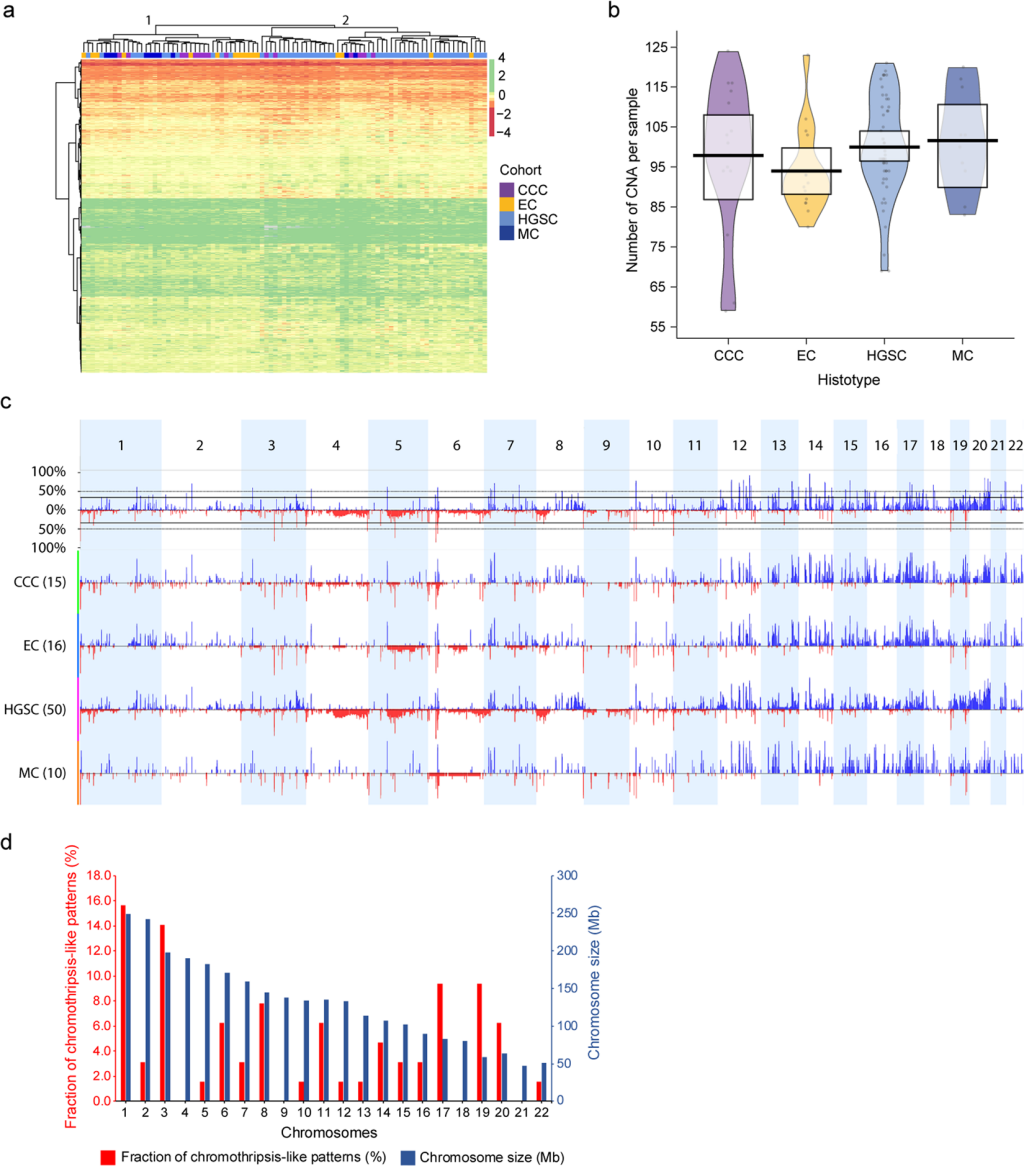
DNA copy number alteration patterns and genomic imbalances. Heatmap (**a**) showing probes (n = 6,651) spanning regions of DNA copy number alterations (CNAs) in at least 35% of the patient samples. All histotypes were distributed across both main clusters (clusters 1 and 2). Moreover, no clear differences in CNA patterns were found in the two main clusters (clusters 1 and 2). Gains are denoted in green color and losses in red color. Gray color denotes missing values (NAs). Canberra distance measure was used to calculate the distance between probes and the Ward method was applied for hierarchical clustering of the histotypes (colored bar at the top of the heatmap). RDI plot (**b**) showing the average number of CNA per patient sample in the respective histotypes, with the greatest mean identified for MC (101.6, range: 83–120) followed by HGSC (100.0, range: 69–121), CCC (97.9, range: 59–124) and EC (94, range: 80–123). All comparisons were non-significant, except for the comparison between EC and HGSC CNAs (Wilcoxon *P* value<0.05). Black open circles distributed horizontally represent raw data probes and the surrounding colored beans depict smoothed densities thereof. Genome-wide frequency plots (**c**) showing CNAs identified in the patient cohort as a whole (top frequency plot) and stratified by histotype (CCC, EC, HGSC, and MC). Chromosomes 1 to 22 are shown in alternating blocks of light blue. Genomic gains are illustrated in blue and genomic losses in red. Bar chart for chromothripsis-like patterns (CTLP) in ovarian carcinoma (**d**) showing the frequency of CTLPs (red). Blue bars show the respective sizes in Megabases (Mb) for chromosomes 1 to 22. Default settings were applied for the CTLPScanner with absolute threshold values for gains and losses set at 0.3. The highest frequency of CTLPs were identified on chromosomes 1, 3, 17 and 19.

Genome-wide profiling of DNA copy number alterations was performed using DNA methylation data for the 91 patient samples with the conumee package in R and the Rank segmentation algorithm in Nexus Copy Number Discovery. In total, 6,651 probes spanned 61 significant CNAs (copy number gains (51/61) and losses (10/61)) in at least 35% of the patient samples (Fig. 3a). Hierarchical clustering of the 6,651 probes stratified the samples into two main clusters (clusters 1 and 2). However, clustering of CNAs was not a good determinate of histotypes classification, as the histotypes were distributed across both clusters. No clear pattern of gains (green) and losses (red) could be seen for specific histotypes.

Among the 61 recurrent CNAs, the average CNA region length was 0.56 ± 0.068 Mb (range=0.7kb-2.25 Mb), while the average number of CNAs per ovarian cancer patient (n = 91) was 98.7 ± 1.44. MC tumors harbored the highest number of CNAs per patient (101.6 ± 4.04, range: 83–120) followed by HGSC (100.0 ± 1.82, range: 69–121), CCC (97.9 ± 4.95, range: 59–124) and EC (94 ± 2.67, range: 80–123) (Fig. 3b). A statistically significant difference in the number of CNAs per patient was however only found between EC and HGSC (Wilcoxon *P* value<0.05). Recurrent loss of genomic content was observed on chromosomal subregions 4q35.2, 5q14.1, 6p22.3, 8p21.3, 10p12.31, 11p15.5, 12p11.23, 12q24.21, 13q21.33, 19q13.32, while recurrent gain was identified on all autosomal chromosomes except for 9, 11 and 19 (Fig. 3c, Supplementary Table 2). In comparison with the other histotypes, CNAs identified in MC generally spanned all autosomal chromosomes rather than focal genomic regions. The recurrent losses on chromosome 4q, 5q and 8p were most prominent in HGSCs, whereas little to no recurrent losses were found for the remaining histotypes. Only recurrent losses on 6p were present in all histotypes. Recurrent gains on chromosomes 19 and 20 were predominantly found in HGSCs, while all other regions of recurrent gains were present in all histotypes. Differential CNAs were identified between histotypes using Nexus Copy Number set at 25%. A total number of 164 CNAs (144 gains, 20 losses) differed between CCC and MC, 208 CNAs (168 gains, 40 losses) between EC and CCC, 145 CNAs (133 gains, 12 losses) between EC and MC, 543 (485 gains, 57 losses) between HGSC and CCC, 660 CNAs (625 gains, 35 losses) between HGSC and EC, 540 CNAs (483 gains, 57 losses) between HGSC and MC.

Genomic instability (chromothripsis-like patterns (CTLP)) was then evaluated using the CTLPScanner with CNA segments identified in ChAMP. CTLPs were defined as more than 20 CNA status changes and an absolute log2 ratio of 0.3 for genomic gains and losses. In total, 64 CTLPs, all of which were CNA gains, were identified in 33/91 (36%) tumor samples. Furthermore, CTLPs were most prevalent on chromosomes 1 (16%), 3 (14%), 17 (9%) and 19 (9%) (Fig. 3d, Supplementary Table 3). On average, the CNA status changed 40 times (range, 20–129 changes) and spanned 50.5 Mb (range, 30–133.3 Mb). The highest number of CTLPs were found in HGSC (n = 46), followed by CCC (n = 8), EC (n = 6), and MC (n = 4). In total, 224 different cancer-related genes were found to span the CTLPs, wherein *MLF1* was most common in 7/64 CTLPs. In addition, well-established cancer-related genes spanning CTLPs included *BRCA1*, *CCNE1*, *TP53* (identified in five CTLPs each), and *ARID1A*, *MYC* and *PIK3CA* (identified in four CTLPs each) (Supplementary Table 3).

### Underexpression was prominent in ovarian carcinoma compared to normal ovarian tissue

**Figure 4:**
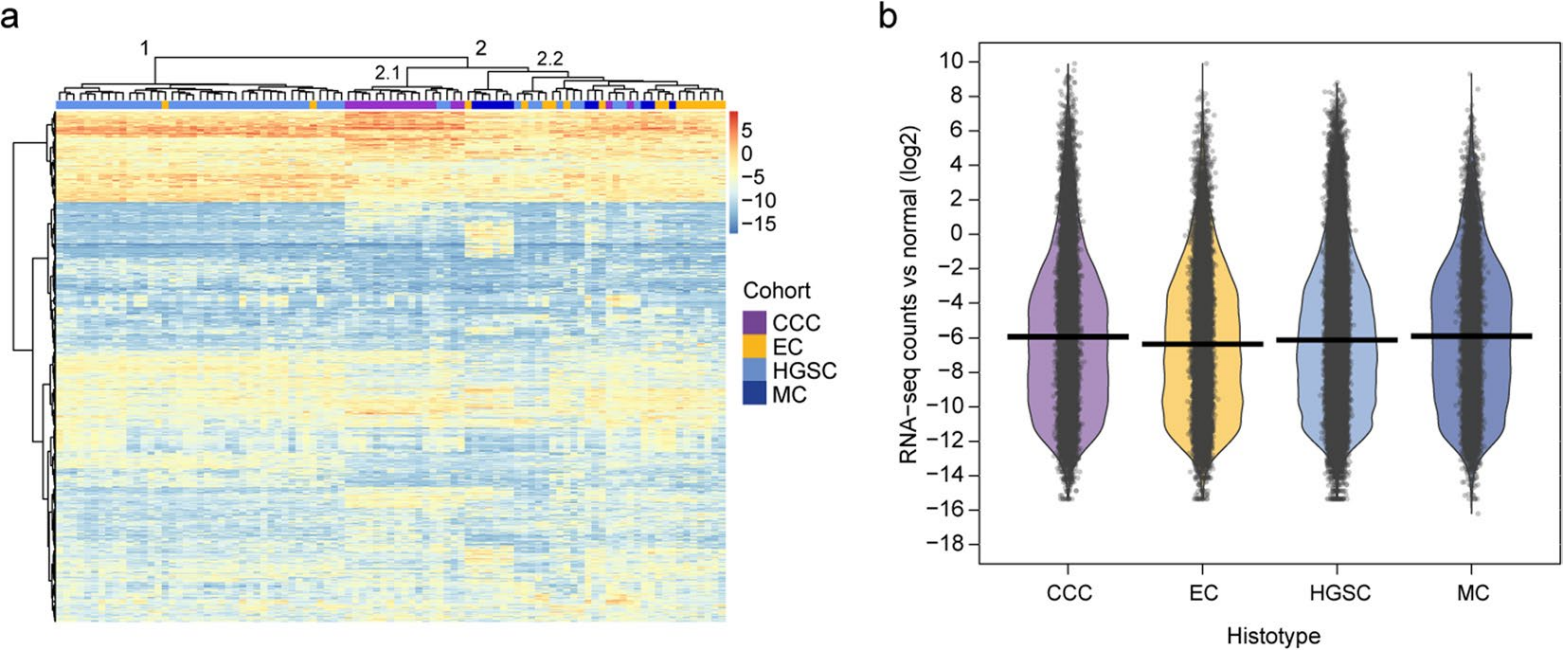
RNA expression analysis. Heatmap (**a**) displaying expression patterns for the 1,000 most variable transcripts across the patient cohort (n = 95). The RNA-seq raw counts (log2 scale) were compared with normal ovarian carcinoma samples downloaded from the Cancer Genome Atlas (TCGA), TCGA-OV data collection. An expression value of log2 ratio>0.58 (*i.e*. 1.5 fold change) was set for overexpression (red) and log2 ratio<−0.58 (*i.e*. 1.5 fold change) for underexpression (blue). In general, the expression levels for ovarian carcinomas were lower in comparison with normal ovarian samples for the 1,000 genes with the highest variance. Canberra distance measure was used to calculate the distance between raw count values. Two main clusters (clusters 1 and 2) were formed using hierarchical clustering (Ward’s method), wherein cluster 1 mainly comprised of HGSC (two EC samples were also included). The majority of CCC samples clustered together in cluster 2.1, whereas cluster 2.2 comprised all histotypes. RDI plot (**b**) for corresponding input data set as in (**a**) displaying the expression differences between histotypes (CCC, EC, HGSC, MC) for the 1,000 most variable genes in terms of variance (RNA-seq counts compared with normal ovarian samples (log2)). Black open circles distributed horizontally represents RNA-seq raw counts and the surrounding colored beans depict smoothed densities thereof. The average values of expression (average expression values of around -6) and bean densities seem rather similar for all histotypes. However, statistical significance was found for all RNA expression comparisons using Wilcoxon test (CCC vs MC: *P* value<0.05; CCC vs HGSC: *P* value<0.001; CCC vs EC, EC vs HGSC, EC vs MC and HGSC vs MC *P* values<0.0001).

The expression profiles for 95 early-stage ovarian carcinomas were analyzed using transcriptome-wide RNA-seq data. Hierarchical clustering, performed using the 1,000 transcripts with the highest variance across the cohort (log2 ratio of ovarian carcinoma compared to normal ovarian tissue), showed that expression was generally lower in ovarian carcinomas compared to normal ovarian samples (Fig. 4a). In addition, the patients were clustered into two main clusters (clusters 1 and 2), wherein cluster 1 was mainly comprised of HGSC samples, whereas cluster 2 contained all histotypes. Samples classified as CCC clustered together, with the exception of two samples in cluster 2.1. Samples classified as EC were distributed over both clusters. The 1,000 transcripts were also clustered into two main clusters (top and bottom clusters), wherein the top cluster comprised genes with overexpression in neoplastic tissue compared with normal ovarian tissue. Interestingly, two genes (AC244035.3 (small nucleolar (sno) RNA) and AL157931.1 (long non-coding (lnc) RNA)) were highly expressed in ovarian carcinoma (all histotypes) with log2 ratios above 4. These genes were also specific for CCC overexpression (wherein all genes had log2 ratios>4) in cluster 2.1, along with two additional genes (AL356277.2 (lncRNA), LINC01320 (lncRNA)). Furthermore, statistically significant differences in expression patterns (RNA-seq raw counts) for the 1,000 most variable transcripts were found between the histotypes (Fig. 4b).

The differential expression analysis identified the highest number of differentially expressed genes (DEGs) between HGSC and CCC, and the lowest number of DEGs between EC and MC (Benjamini-Hochberg adjusted *P* value<0.05). More specifically, 3,061 DEGs (1,447 over-, 1,614 underexpressed) between CCC and MC, 2,764 DEGs (1,476 over-, 1,288 underexpressed) between EC and CCC, 792 DEGs (376 over-, 416 underexpressed) between EC and MC, 4,990 DEGs (2,882 over-, 2,108 underexpressed) between HGSC and CCC, 1,430 DEGs (736 over-, 694 underexpressed) between HGSC and EC, and 3,685 DEGs (2,037 over-, 1,648 underexpressed) between HGSC and MC. Overall, 212 genes and 139 genes were overexpressed and underexpressed, respectively, in all HGSC comparisons. In the EC comparisons, 57 genes and 13 genes were overexpressed and underexpressed, respectively, and in the MC comparisons 229 genes and 82 genes were overexpressed and underexpressed, respectively. Ingenuity Pathway Analysis (IPA) revealed an association with cancer including top biological processes related to molecular and cellular functions such as cellular movement, molecular transport, lipid metabolism, and cell death and survival (Supplementary Table 4).

### Integrative genomics profiling identifies molecular features associated with early-stage ovarian carcinoma histotypes

Integrative analyses were performed to assess the effect of DNA methylation and CNA patterns on gene expression. Cluster-of-cluster analysis (COCA) was then performed using the coca R package with the RNA-seq, DNA methylation and CNA data (Supplementary Fig. 2)^12^. Two COCA clusters (1 and 3) corresponded with purely HGSC samples, whereas cluster 2 comprised all histotypes, and cluster 3 a mix of CCC, EC and HGSC samples. Putative genetic markers in ovarian carcinoma were further identified. A total of 49 genes were found to be either overexpressed, hypomethylated and showed genomic gain (46/49 putative oncogenes), or underexpressed, hypermethylated and showed genomic loss (3/49 putative tumor suppressor genes) when comparing the histotype groups (CCC vs MC, EC vs CCC, EC vs MC, HGSC vs CCC, HGSC vs EC, HGSC vs MC) (Table 1). The highest number of deregulated genes was found in HGSCs compared with CCCs (n = 23). *LINC00578* was found to be overexpressed, hypomethylated and showed genomic gain in all HGSC comparisons, and *CLMN* was overexpressed, hypomethylated and showed genomic gain in two comparisons (EC vs CCC, HGSC vs CCC). No genes were found to be deregulated in all three methods when comparing EC with MC. Overexpression, hypomethylation and genomic gain was demonstrated for *CCNE1* on chromosome 19 in the HGSC vs EC comparison (Supplementary Fig. 3, Table 1). Further genomic gains was also highlighted for *COL14A1* and *MTBP* and genomic loss for *ELP3* on chromosome 8. Seventeen of 49 biomarkers were found in enhancer regions (Table 1). The presence of mutations in the identified putative oncogenes and tumor suppressor genes was examined (Supplementary Table 5). The mutation frequency in the *MTBP* gene was the highest with a frameshift insertion in 9.5% of the patient samples, whereas the remaining mutation frequencies were relatively low (1.1–2.1%). Further putative oncogenes/tumor suppressor genes were altered with at least two mechanisms, *i.e*. overexpressed and hypomethylated, overexpressed and showed genomic gain, underexpressed and hypermethylated, or underexpressed and showed genomic loss (Benjamini-Hochberg adjusted *P* value<0.05, Supplementary Table 6, Supplementary Fig. 4).

**Table 1:** Integrated RNA sequencing, DNA methylation and DNA copy number alteration(CNA) analysis.

Gene symbol	Genomic region	Enhancer	Gene expression^a^	DNA methylation^b^	DNA copy number alteration^c^
**CCC case vs MC control**					
*FAM20A*	17q24.2-q24.3	NA	overexpressed	hypomethylated	genomic gain
*LAMB1*	7q22.3-q31.1	NA	overexpressed	hypomethylated	genomic gain
**EC case vs CCC control**					
*CLMN*	14q32.13	NA	overexpressed	hypomethylated	genomic gain
**HGSC case vs CCC control**					
*CACNA1A*	19p13.13	yes	overexpressed	hypomethylated	genomic gain
*CACNB1*	17q12	yes	overexpressed	hypomethylated	genomic gain
*CELF4*	18q12.2	NA	overexpressed	hypomethylated	genomic gain
*CLMN*	14q32.13	yes	overexpressed	hypomethylated	genomic gain
*COL14A1*	8q24.12	yes	overexpressed	hypomethylated	genomic gain
*EBF4*	20p13	NA	overexpressed	hypomethylated	genomic gain
*EHF*	11p13	yes	overexpressed	hypomethylated	genomic gain
*HMGA2*	12q14.3	yes	overexpressed	hypomethylated	genomic gain
*IDH3B*	20p13	NA	overexpressed	hypomethylated	genomic gain
*KCNMB2*	3q26.32	NA	overexpressed	hypomethylated	genomic gain
*LINC00578*	3q26.32	yes	overexpressed	hypomethylated	genomic gain
*MAPK4*	18q21.1-q21.2	NA	overexpressed	hypomethylated	genomic gain
*MEIS2*	15q14	NA	overexpressed	hypomethylated	genomic gain
*MTBP*	8q24.12	NA	overexpressed	hypomethylated	genomic gain
*MYLK2*	20q11.21	NA	overexpressed	hypomethylated	genomic gain
*NRSN2*	20p13	NA	overexpressed	hypomethylated	genomic gain
*PDYN*	20p13	yes	overexpressed	hypomethylated	genomic gain
*PPP1R1B*	17q12	yes	overexpressed	hypomethylated	genomic gain
*PROKR2*	20p12.3	NA	overexpressed	hypomethylated	genomic gain
*RASSF2*	20p13-p12.3	NA	overexpressed	hypomethylated	genomic gain
*RPL22L1*	3q26.2	NA	overexpressed	hypomethylated	genomic gain
*TP63*	3q28	NA	overexpressed	hypomethylated	genomic gain
*ELP3*	8p21.1	NA	underexpressed	hypermethylated	genomic loss
**HGSC case vs EC control**					
*AARD*	8q24.11	NA	overexpressed	hypomethylated	genomic gain
*CCNE1*	19q12	NA	overexpressed	hypomethylated	genomic gain
*CTCFL*	20q13.31	NA	overexpressed	hypomethylated	genomic gain
*LINC00578*	3q26.32	yes	overexpressed	hypomethylated	genomic gain
*LINC01532*	19q12	NA	overexpressed	hypomethylated	genomic gain
*RBM38*	20q13.31	yes	overexpressed	hypomethylated	genomic gain
*RSPO4*	20p13	NA	overexpressed	hypomethylated	genomic gain
*UQCRFS1*	19q12	NA	overexpressed	hypomethylated	genomic gain
*URI1*	19q12	NA	overexpressed	hypomethylated	genomic gain
*PDE8B*	5q13.3	NA	underexpressed	hypermethylated	genomic loss
**HGSC case vs MC control**					
*ANKS1B*	12q23.1	yes	overexpressed	hypomethylated	genomic gain
*COLEC10*	8q24.12	yes	overexpressed	hypomethylated	genomic gain
*EGFEM1P*	3q26.2	NA	overexpressed	hypomethylated	genomic gain
*KCNMB2-AS1*	3q26.32	NA	overexpressed	hypomethylated	genomic gain
*LINC00578*	3q26.32	yes	overexpressed	hypomethylated	genomic gain
*MYEF2*	15q21.1	NA	overexpressed	hypomethylated	genomic gain
*PDYN*	20p13	NA	overexpressed	hypomethylated	genomic gain
*RBFOX1*	16p13.3	yes	overexpressed	hypomethylated	genomic gain
*SNAP25*	20p12.3-p12.2	NA	overexpressed	hypomethylated	genomic gain
*STON2*	14q31.1	yes	overexpressed	hypomethylated	genomic gain
*SULF1*	8q13.2-q13.3	NA	overexpressed	hypomethylated	genomic gain
*TSHR*	14q31.1	yes	overexpressed	hypomethylated	genomic gain
*TGFBR2*	3p24.1	NA	underexpressed	hypermethylated	genomic loss

The table shows putative oncogenes and tumor suppressor genes that were found to be deregulated using three different methods (RNA-seq, DNA methylation, CNA analyses). Generally, the genes were overexpressed, hypomethylated and showed genomic gain. A few genes were also underexpressed, hypermethylated and showed genomic loss. No biomarkers were found to be deregulated in all three methods when comparing EC with MC.

^a^Gene expression log2 ratios greater than 0.58 (i.e. 1.5 fold change) are indicated by overexpression and less than -0.58 (i.e. 1.5 fold change) by underexpression.

^b^DNA methylation delta beta values greater than 0.2 are indicated by hypermethylation and less than -0.2 by hypomethylation.

^c^DNA copy number alteration values greater than 0.3 are indicated by genomic gain and less than -0.3 by genomic loss.

## Discussion

Ovarian cancer is a rare disease with 541 patients diagnosed in 2016 in Sweden. In comparison, 8,923 female breast cancer patients were diagnosed in the same year in Sweden^13^. Early-stage ovarian carcinoma is less frequently diagnosed in comparison with later stages (stage I + II: 36%, stage III + IV: 62%)^14^. Hence, large ovarian carcinoma patient cohorts, especially early-stage cohorts, are difficult to achieve. Previous studies have primarily focused on single histotypes, *e.g*. HGSC in TCGA ovarian carcinoma cohort, and CCC in an epigenome-wide analysis of CCC-specific DNA methylation patterns^4,15^. Therefore, it may be difficult to compare between different histotype-specific studies due to *e.g*. differences in patient diagnosis and treatment protocols, and experimental conditions and technologies used. Moreover, few ovarian carcinoma studies have integrated omics-wide analyses, *e.g*. the integration of high-throughput technologies of genetic, epigenetic and transcriptomic alterations. A recent report classified DNA methylation patterns associated with histotypes (70 HGSC, 6 LGSC, 30 serous low malignant potential (LMP) carcinomas, 16 MC, 33 EC, 7 CCC) in all stages (I-IV)^7^. Furthermore, a subset of the tumors were also analyzed using CNA analysis (47/162 samples, 180 K-feature aCGH assay), and gene expression analysis using NanoString assay (61/162 samples) but was limited to only HGSC samples. In total, only 13 tumor samples (HGSC) were analyzed using all three methods. Hence, the current study is, to the best of our knowledge, the first to present a comprehensive genome- and transcriptome-wide analysis of DNA methylation, CNA and RNA-seq data (on the same patient cohort) from primary invasive early-stage ovarian carcinoma samples (n = 96) constituting multiple histotypes (CCC, EC, HGSC, MC).

It is well known that aberrant DNA methylation (*e.g*. hypomethylation/hypermethylation of genes and gene regulatory elements) affects gene expression^16^. Here, the DNA methylation analysis revealed a higher relative distribution of unmethylated CpG sites in promoters, enhancers and exons, as well as in CpG islands and shores. It has been shown that not only promoter and gene body methylation, but also enhancer methylation can lead to altered gene expression, highlighting the importance of examining methylation patterns in other genomic regions outside of promoter and gene body regions^17^. Unique DNA methylation patterns were revealed for each histotype, wherein CCC had the highest mean distribution of methylated probes and a higher relative distribution of hypermethylated DMPs in comparison with the other histotypes (EC, HGSC and MC). HGSC showed the lowest mean distribution of methylated probes, and HGSC and MC were generally hypomethylated compared to CCC and EC. These findings are in line with previous reports showing promoter hypermethylation in CCC and hypomethylation in HGSC, but DNA methylation patterns for MCs are largely unknown^6^. Hierarchical clustering of the 1000 most variable probes revealed the heterogeneous nature of HGSC, where one cluster contained only HGSC samples and the other cluster was a composite of all four histotypes.

The DNA methylation data could better classify the patients according to the histotypes compared to the CNA data. This may be explained by the detection of non-cancer related CNAs due to genomic instability, which may in turn affect accurate histotype classification. Moreover, tumor-specific CNAs may be diminished by contamination of normal cells and/or intratumor heterogeneity. In Nexus Copy Number, the default for recurrent CNAs is 25% and was used to compare identified CNAs in different histotypes. However, a cutoff of 35% was chosen to reduce the number of significant CNA changes identified in the ovarian carcinoma cohort. Interestingly, the highest average number of CNAs per patient was found for MCs. However, no significant statistical difference was found when comparing MC with the other histotypes. Little has previously been reported for CNA changes in MC tumors. One report demonstrated low numbers of CNAs in MC compared with the other main histotypes, however the MC patient cohort (n = 14) was relatively small and no information regarding tumor stage was given^8^. The discrepancy between the studies may be explained by *e.g*. contamination of normal cells and/or intra-tumor heterogeneity affecting the detection of CNAs. The second highest average number of CNAs per patient was identified in HGSCs, which is in line with previous reports demonstrating a high frequency of CNA gains and losses in HGSCs^18^. Moreover, consistent with previous reports on genomic instability, the highest number of CTLPs was also revealed in HGSCs^18^. MC tumors showed the lowest number of CTLPs which may be explained by the identification of CNA changes in specific chromosomal regions, compared to rather widespread CNA patterns in the other histotypes.

In agreement with the DNA methylation data, RNA expression analysis was able to classify the different histotypes, thereby demonstrating significant differences between the histotypes. However, differences between the histotypes were more evident in the DNA methylation heatmap. To the best of our knowledge, we are the first to report high expression of snoRNA AC244035.3 and lncRNA AL157931.1 in ovarian carcinoma (all histotypes). Furthermore, these genes and additionally lncRNA AL356277.2 and lncRNA LINC01320 have not previously been reported to be highly expressed within CCC. The highest number of DEGs were identified when comparing HGSC with CCC, and the lowest number when comparing EC with MC (Benjamini-Hochberg adjusted *P* value<0.05). The low number of DEGs between EC and MC may be explained by the fact that EC may comprise MC differentiation in the epithelial structure. Few studies have previously examined differences in gene expression patterns between the ovarian carcinoma histotypes, particularly in early-stages^19,20^. In a previous study using the same cohort presented here, we identified novel histotype-specific mutation profiles comprised of recurrent deleterious mutations (present in at least 30% of patients within each histotype (CCC, EC, HGSC, MC)) in 38 genes. Moreover, the highest mutation frequency of *e.g. TP53* was found in early-stage HGSC^21^.

Advances in molecular biology have shown that mechanisms affecting aberrant gene expression profiles leading to cancer initiation and progression cannot be explained by genetic alterations (mutations, DNA CNAs, inversions, insertions or translocations) alone. Further changes, such as epigenetic aberrations also influence gene expression, highlighting the importance of integrative approaches in the identification of robust biomarkers^22^. In the current study, novel putative oncogenes and tumor suppressor genes (n = 49) associated with ovarian carcinoma histotypes were identified using an integrative approach with DNA methylation, CNA and RNA-seq data. The majority of the identified genes were found to be overexpressed, hypomethylated and showed DNA gain. The highest number of putative oncogenes/tumor suppressor genes were found when comparing HGSC with CCC, which is not surprising since this comparison also generated the highest number of DEGs, and the second highest number of DMPs and CNAs, respectively. For 19 of the 49 putative oncogenes/tumor suppressor genes, a previous connection with ovarian carcinoma has been reported. For example, *LAMB1* (CCC vs MC comparison), which encodes an extracellular matrix glycoprotein involved in cell adhesion and migration, was reported to be differentially expressed across a cohort of HGSC, EC and CCC tumors^19^. Moreover, high expression of *HMGA2* (HGSC vs CCC comparison), a transcription factor constituting an important part of the enhancesome, was reported in the proliferative HGSC subtype of the TCGA ovarian carcinoma cohort^4^. *HMGA2* was upregulated in both early- and late-stage HGSC^23^. Multiple studies have associated *CCNE1* (HGSC vs EC comparison), which is known to promote accelerated S phase entry and thereby promote genetic instability, with amplification in HGSC, which may also contribute to chemotherapy resistance^24,25^. The *URI1* gene (HGSC vs EC comparison), involved in ubiquitination and transcription, spans the same genomic region as *CCNE1*, and has also been reported to be amplified in ovarian carcinoma and may contribute to tumorigenesis^26^. For the remaining identified putative oncogenes/tumor suppressor genes (30/49), no previous connection has been reported in connection with ovarian carcinomas, but may be known to be involved in tumorigenesis of other cancer types *e.g. MTBP* (HGSC vs CCC comparison), which interacts with *MYC* to promote tumorigenesis, has been associated with overexpression in triple-negative breast cancer^27,28^.

To conclude, we have provided a comprehensive overview of histotype-specific molecular aberrations on the DNA and RNA level in early-stage ovarian carcinomas (n = 96). More specifically, we identified methylation patterns, CNAs and aberrant RNA expression relating to individual early-stage ovarian carcinoma histotypes (CCC, EC, HGSC, and MC). We integrated these data to identify novel putative oncogenes and tumor suppressor genes, which to the best of our knowledge have not previously been associated with early-stage ovarian carcinoma histotypes. Advantages of the study comprise the involvement of patients from multiple subtypes (4/5 of the main histotypes), *i.e*. not only the largest histotype group HGSC, but also the smaller and less studied histotypes such as MC. Further, the patients included in the study were subjected to the same diagnostic and treatment procedures according to national guidelines (staging and accurate debulking cytoreductive surgery), thereby reducing possible biases and enabling easier comparison across histotypes and molecular levels. Although a fairly large patient cohort, the main drawback is the difficulty in achieving even larger patient cohorts, especially for early-stage disease in the rarer histotype groups. Moreover, it is difficult to compare the histotypes with their respective true normal tissues since it is currently not known from where all histotypes originate, as well as few publicly available datasets on normal gynecological tissues, *e.g*. TCGA has publicly available data for normal ovarian tissue, but not for fallopian tube tissue. Future functional analyses need to be performed to confirm the role of the putative oncogenes and tumor suppressor genes in ovarian carcinoma. Moreover, analyses on the protein level are needed to validate histotype-specific patterns using *e.g*. immunohistochemistry.

## Methods

### Patients and tumor samples

A total of 96 early-stage (stage I and II) primary invasive ovarian carcinoma patients (diagnosed between 1994 and 2006) were included in the cohort. Fresh-frozen tumor samples were obtained from the tumor bank at the Sahlgrenska University Hospital Oncology lab (Gothenburg, Sweden). The tumors were reclassified to current WHO criteria for ovarian carcinoma histotypes by board certified pathologists using corresponding full-face formalin-fixed paraffin-embedded (FFPE) samples obtained from the Department of Clinical Pathology at Sahlgrenska University Hospital^29^. The reclassified tumor samples comprised 17 CCC, 17 EC, 51 HGSC and 11 MC. Additional clinicopathological information for the cohort were obtained from the Cancer Registry at the National Board of Health and Welfare (Stockholm, Sweden) and the National Quality Registry at the Regional Cancer Center West (Gothenburg, Sweden) (Table 2, Supplementary Table 7). National treatment guidelines with protocols for standard surgery procedures (staging and adequate debulking cytoreductive surgery) were followed for all patients. The study was performed in accordance with the Declaration of Helsinki and approved by the Regional Ethical Review Board (Gothenburg, Sweden; case number 767–14). Moreover, the Regional Ethical Review Board further approved a waiver of written consent to use the tumor specimens. The percentage of neoplastic cells was assessed in all samples using touch preparation imprints stained with May-Grünwald Giemsa (Chemicon). Highly representative tumor samples comprising at least 50% neoplastic cell content were included in subsequent analyses.

**Table 2:** Clinicopathological features for the patient cohort (n = 96).

	Number of patients (%)				*P* value
	CCC (n = 17)	EC (n = 17)	HGSC (n = 51)	MC (n = 11)	
**Patient age**					0.95
Mean	63	64	63	61	
Range	42–84	25–83	32–86	39–80	
**Overall Survival**					0.45
0–2 y	2 (12)	1 (6)	2 (4)	3 (27)	
2–5 y	3 (18)	5 (29)	17 (33)	2 (18)	
5–10 y	7 (41)	5 (29)	19 (37)	3 (27)	
>10 y	5 (29)	6 (35)	13 (25)	3 (27)	
**Cause of death**					**0.01**
Ovarian carcinoma	10 (59)	3 (18)	33 (65)	2 (18)	
Other cancer	0 (0)	3 (18)	7 (14)	3 (27)	
Other	6 (35)	6 (35)	5 (10)	4 (36)	
Not available	1 (6)	0 (0)	0 (0)	0 (0)	
Alive	0 (0)	5 (29)	6 (12)	2 (18)	
**Stage**					0.15
I	14 (82)	11 (65)	29 (57)	9 (82)	
II	3 (18)	6 (35)	22 (43)	2 (18)	
**Tumor grade EC**					NA
FIGO grade I	NA	2 (12)	NA	NA	
FIGO grade II	NA	9 (53)	NA	NA	
FIGO grade III	NA	6 (35)	NA	NA	
**CA125**					0.068
<35	6 (35)	7 (41)	9 (18)	5 (45)	
35–65	1 (6)	0 (0)	29 (57)	2 (18)	
>65	10 (59)	10 (59)	13 (25)	4 (36)	
Not available	0 (0)	0 (0)	0 (0)	0 (0)	
**Ploidy**					0.095
Near diploid	1 (6)	7 (41)	15 (29)	2 (18)	
Aneuploid	16 (94)	9 (53)	36 (71)	8 (73)	
Not available	0 (0)	1 (6)	0 (0)	1 (9)	
**Chemotherapy**					NA
Yes	17 (100)	17 (100)	49 (96)	11 (100)	
No	0 (0)	0 (0)	0 (0)	0 (0)	
Not available	0 (0)	0 (0)	2 (4)	0 (0)	

Significant values (*P* value<0.05) are marked in bold.

### DNA methylation analysis

DNA was extracted from fresh-frozen tumor tissues from 91/96 tumors (15 CCC, 16 EC, 50 HGSC and 10 MC) that had sufficient tumor material remaining in the tumor bank using the Wizard Genomic DNA extraction kit (Promega), and purified with phenol‐chloroform purification. All samples had 260/280 ratios greater than 1.8 as measured with Nanodrop ND-1000 spectrophotometer (Nanodrop Technologies). The purified genomic DNA was analyzed with Illumina Infinium MethylationEPIC BeadChips (MethylationEPIC, v. 1.0; genomic build, v. 37) at the SNP&SEQ Technology Platform (Uppsala, Sweden).

The DNA methylation analyses were performed in R/Bioconductor (v. 3.6.0). Raw data were processed using the R package ChAMP (v. 2.14.0)^30,31^. More specifically, raw intensity data were generated from IDAT files and subjected to ChAMP default filtering steps (*e.g*. probes with detection *P* value>0.01, non-CpG probes, single nucleotide polymorphism (SNP)-related probes presented elsewhere^32^, and probes located on chromosome X and Y, were removed), resulting in 694,299 CpG sites. The BMIQ normalization method was used to adjust for differences in probe type (probe I/II) and corrections were made for batch effects (array, slide) using the myCombat function in ChAMP (n = 679,259 CpG sites) including the sva package (v. 3.32.1) (Supplementary Fig. 1)^33^. Probe information including *e.g*. chromosome, gene, type of genomic regions (promoter region (200bp-1500bp upstream of transcriptional start sites, 1^st^ exon, 5′ untranslated region (5′ UTR)), gene body, 3′UTR, intergenic region (IGR) and exon) and regions surrounding CpG islands (CpG islands (genomic region >200 bp long with >50% G and C nucleotide content), CpG shores (0–2 kb from CpG islands), CpG shelves (2–4 kb from CpG islands) and open sea (>4 kb from CpG islands)) was retrieved using probe.features in ChAMP, and enhancer information was added from the methylation EPIC manifest file (MethylationEPIC_v-1-0_B4_ManifestFile.csv). Beta value density plots were generated before and after normalization, as well as after batch correction to examine possible outliers. The 1000 most variable probes in the cohort were identified by ordering the batch corrected probes according to the greatest variance. Histotype-specific DMPs were identified using the limma package (v. 3.40.2) with Benjamini-Hochberg adjusted *P* value<0.05 and >1.5 fold change, and a Venn diagram was constructed to visualize unique and overlapping DMPs between the histotypes^34^.

### DNA copy number alteration analysis

Unsegmented CNA data for single probe resolution was extracted from the batch corrected DNA methylation data (n = 91 patients) using the conumee package (v. 1.18.0) in R^35^. The CNA data was normalized using 52 control samples from healthy individuals in the CopyNumber450kData package (v. 1.8.0.) to correct for probe and sample bias^36^. Since the available control samples were from the 450k array, only common probes on the EPIC and 450k arrays could be evaluated, yielding 352,016 probes. Probe level, normalized CNA data was used as input to Nexus Copy Number (BioDiscovery, v. 7.5). CNAs were called based on the Rank segmentation algorithm (significance threshold 1.0E-5, maximum contiguous probe spacing 1000 Kbp, minimum number of probes per segment 3), with log2 ratio thresholds for homozygous loss/deletion, heterozygous loss, gainset at ≤ −1, <−0.3, and >+0.3, respectively. Significant CNAs were below *P* value 0.05 and the differential threshold were set at 35% (*i.e*. the genetic aberrations were present in at least 35% of the tumor samples). DNA copy number variations (CNVs, *i.e*. 100% coverage between genomic regions and previously reported CNAs in the human genome) were further removed (n = 30). The CNA function in ChAMP was used to segment the CNA data. The CNA segments for each tumor sample were further evaluated for genomic instability related to chromothripsis-like patterns (CTLPs). CTLPs were detected using the web-based CTLPScanner (http://cgma.scu.edu.cn/CTLPScanner/) with default settings (*e.g*. ≥20 copy number aberration status change times, ≥8 log10 of likelihood ratio, ≥0.3 log2 ratio threshold for genomic gains, ≤-0.3 log2 ratio threshold for genomic losses)^37,38^. Known cancer genes within chromothripsis regions were identified using the Catalogue Of Somatic Mutations In Cancer (COSMIC)^39^.

### Whole-transcriptome RNA sequencing analysis

Aligned reads from NCBI Gene Expression Omnibus (http://www.ncbi.nlm.nih.gov/geo/) under accession number GSE101108 were used for the RNA-seq analysis. One tumor sample (HGSC), had been removed due to poor mapping quality leading to an RNA-seq cohort of 95 samples. The RNA-seq raw counts were converted to log2 scale and compared with the mean of normal ovarian samples (n = 30) downloaded from the Cancer Genome Atlas (TCGA), TCGA-OV data collection^40^. The normal ovarian carcinoma samples were processed in the same manner as the cohort RNA-seq raw counts^21^. The 1,000 most variable transcripts across the cohort were identified by ordering the transcripts according to the greatest variance. Differentially expressed genes (DEGs) between different histotypes were identified using DESeq. 2 (v. 1.14.0) in R/Bioconductor^41^. Significant DEGs were set to Benjamini-Hochberg adjusted *P* value<0.05, and overexpression was set to log2 ratio>0.58 and underexpression to log2 ratio<−0.58. The molecular functions of the DEGs were examined using Ingenuity Pathway Analysis (IPA, Ingenuity Systems, Redwood City, USA). Mutations were identified using the Genome Analysis Toolkit (GATK) Best Practices protocol, subsequently annotated with ANNOVAR, and filtered with the 1000 Genomes Project dataset and dbSNP, as previously described^21,42–44^.

### Integrative RNA sequencing, DNA methylation and DNA copy number alteration analyses

Differentially expressed genes between histotypes were used in the integrative analysis. The differentially methylated probe (DMP) function in ChAMP was used to identify statistically significant DMPs between histotypes (Benjamini-Hochberg adjusted *P* value<0.05). Genes spanning differential CNAs between histotypes were identified using Nexus Copy Number Discovery with a *P* value cutoff at 0.05 and a differential threshold set at 25% (all CNVs were removed). For each dataset (RNA-seq, DNA methylation, DNA copy number alteration), a comparative analysis was performed between the following histotypes: CCC vs MC, EC vs CCC, EC vs MC, HGSC vs CCC, HGSC vs EC, and HGSC vs MC. The comparative analyses were then integrated to identify putative oncogenes (1. overexpressed, hypomethylated, and CNA gain, 2. overexpressed and hypomethylated or 3. overexpressed and CNA gain) and tumor suppressors (1. underexpressed, hypermethylated, and CNA loss, 2. underexpressed and hypermethylated or 3. underexpressed and CNA loss). CNA plots for chromosomes 1 to 22, and zoom-ins on specific chromosomes were generated with the conumee package, with copy number loss and copy number gain. Cluster-of-cluster analysis (COCA) integrating RNA-seq, DNA methylation and CNA data (same input data as for the hierarchical clustering) was performed using the coca R package (v. 1.0.2)^12^.

### Statistical analysis

The statistical analyses were performed in R/Bioconductor using two-sided *P* values<0.05. Heatmaps were constructed using pheatmap (v. 1.0.12)^45^. The Ward’s method was used for the hierarchical clustering of histotypes and Canberra distance measure was used to calculate the distance between different samples to examine the similarity between two samples. The yarr package (v. 0.1.5) was used to compile an RDI (Raw data, Descriptive, Inference statistics) plot to visualize the differences in DNA methylation and RNA-seq levels for the 1000 most variable transcripts/probes between the histotypes^46^.

## Supplementary information

Supplementary Figures and Tables 4, 5, and 7.

Supplementary Table 1.

Supplementary Table 2.

Supplementary Table 3.

Supplementary Table 6.

## Data Availability

The datasets analyzed in this study can be found in the NCBI Gene Expression Omnibus (http://www.ncbi.nlm.nih.gov/geo/) (GSE40744).
