# Supplementary material for: Integrative genomics approach identifies molecular features associated with early-stage ovarian carcinoma histotypes
Source: Sci Rep. 2020 May 14;10:7946. doi: 10.1038/s41598-020-64794-8 (PMC7224294; doi:10.1038/s41598-020-64794-8)
Supplement: Supplementary file 1 — Supplementary Figures and Tables 4, 5, and 7. [file 41598_2020_64794_MOESM1_ESM.pdf]

Integrative genomics approach identifies molecular features associated with early-stage ovarian carcinoma histotypes

Hanna Engqvist[1*], Toshima Z. Parris[1], Jana Biermann[1], Elisabeth Werner Rönnerman[1,2], Peter Larsson[1], Karin Sundfeldt[3], Anikó Kovács[2], Per Karlsson[1‡], Khalil Helou[1‡]

[1]Department of Oncology, Institute of Clinical Sciences, Sahlgrenska Cancer Center, Sahlgrenska Academy at University of Gothenburg, Gothenburg, Sweden, [2]Sahlgrenska University Hospital, Department of Clinical Pathology, Gothenburg, Sweden and [3]Department of Obstetrics and Gynecology, Institute of Clinical Sciences, Sahlgrenska Cancer Center, Sahlgrenska Academy at University of Gothenburg, Gothenburg, Sweden.

‡P. Karlsson and K. Helou contributed equally to this article.

Correspondence: Hanna Engqvist, Department of Oncology, University of Gothenburg, Box 425, SE-40530 Gothenburg, Sweden; Phone: (+46) 31 7866751; E-mail: hanna.engqvist@gu.se. (*)

SUPPLEMENTARY MATERIAL

SUPPLEMENTARY FIGURES

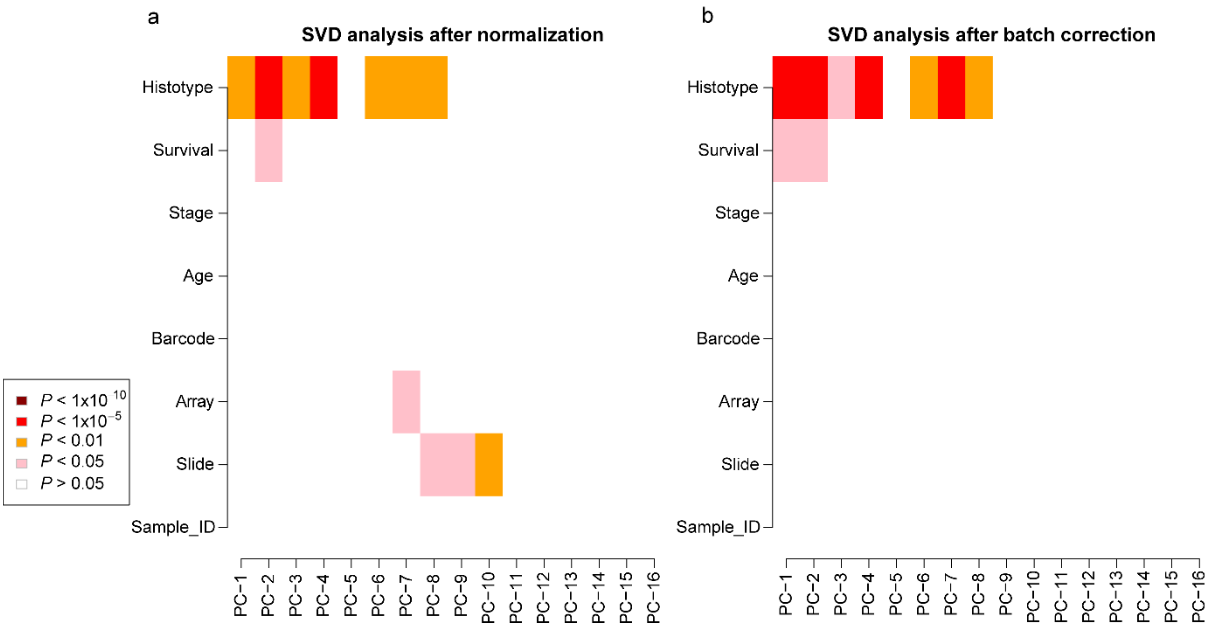

Supplementary Figure 1. Singular value decomposition (SVD) analysis to evaluate differences of biological and technical factors. SVD plots after normalization (a) and after correction of batch effects (b) demonstrating correction for array and slide.

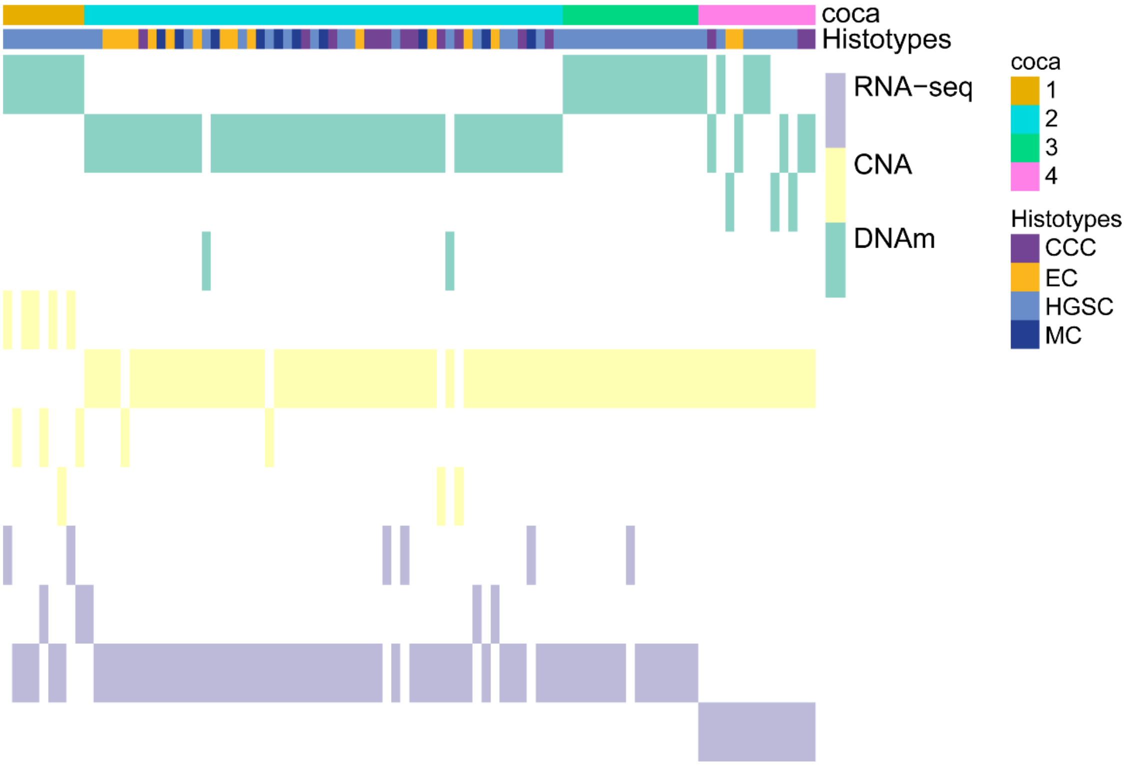

Supplementary Figure 2. Cluster-of-cluster analysis plot showing integrated RNA-seq, DNA methylation and DNA CNA data.

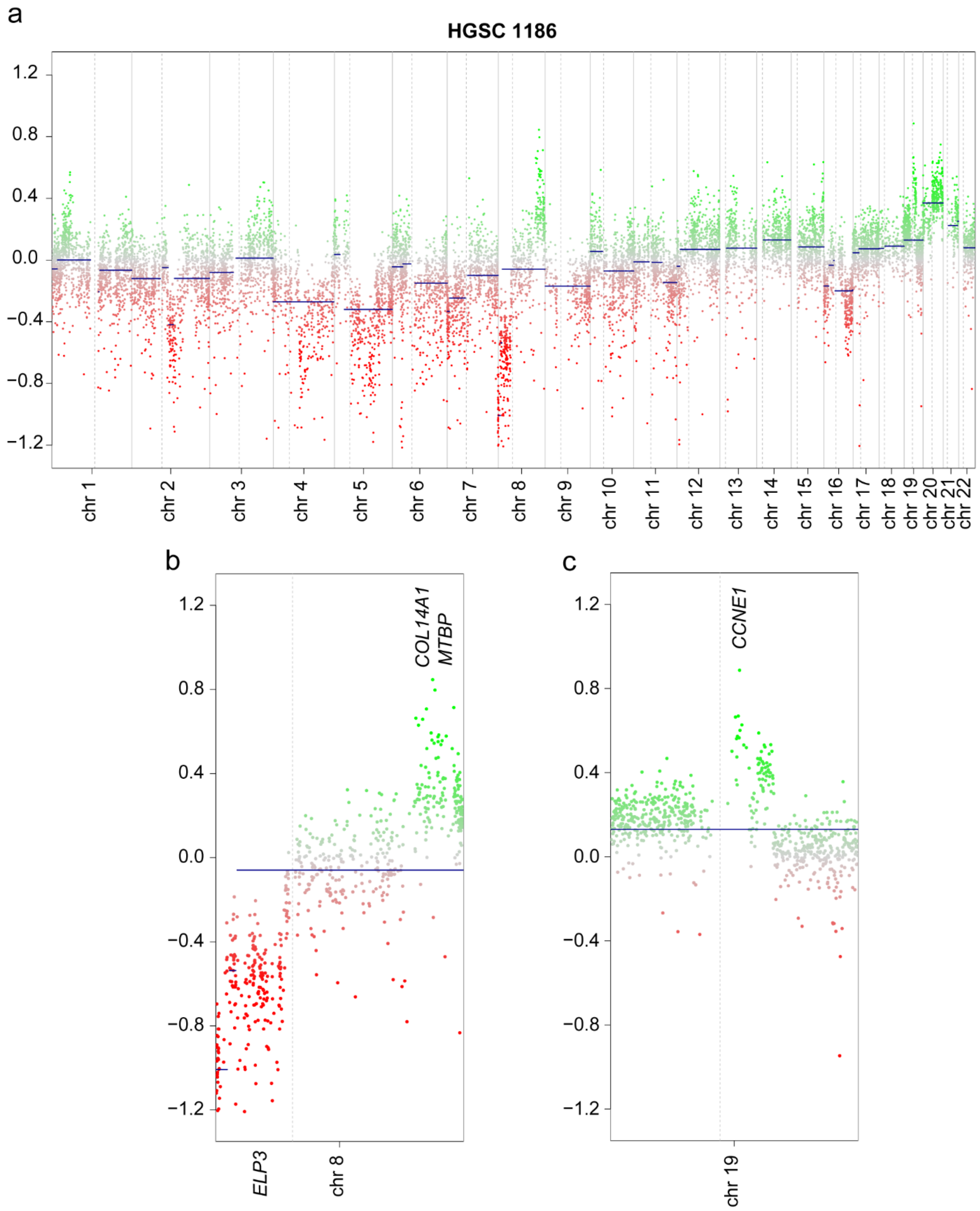

Supplementary Figure 3. Integrative genomic and transcriptomic analyses identifies aberrant genes across histotypes. CNA plot generated using the conumee package for patient sample OV1186 of HGSC histotype showing the genomic profile for chromosomes 1-22 with genomic gain depicted in green and genomic loss depicted in red (a). CNA plots zoom-ins on chromosome 8 showing genomic gains for *COL14A1* and *MTBP*, as well as genomic loss for *ELP3* (b), and on chromosome 19 showing genomic gain for *CCNE1* (c).

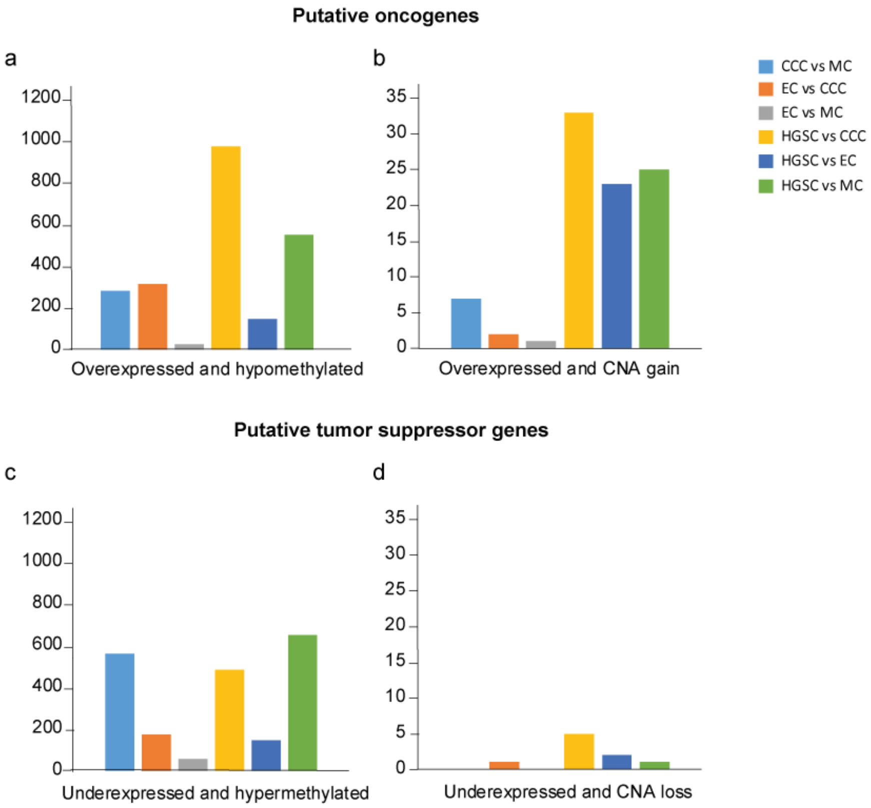

Supplementary Figure 4. Number of genes that were altered by at least two mechanisms, *i.e.* altered gene expression (over-/underexpressed), DNA methylation modulation (hyper-/hypomethylated) or DNA CNA (genomic gain/loss).

SUPPLEMENTARY TABLES

Supplementary Tables 1-3 and 6 are uploaded separately as excel files due to their big sizes.

Supplementary Table 4. Ingenuity Pathway Analysis (IPA) for differentially expressed genes (DEGs) between histotype comparisons (CCC vs MC, EC vs CCC, EC vs MC, HGSC vs CCC, HGSC vs EC, and HGSC vs MC).

Histotype comparison	Top molecular and cellular functions	*P* value	Molecules
CCC vs MC	Lipid Metabolism	8.79E-04 - 1.34E-12	293
	Small Molecule Biochemistry	8.79E-04 - 1.34E-12	324
	Vitamin and Mineral Metabolism	7.19E-04 - 1.37E-11	107
	Cellular Movement	7.83E-04 - 1.25E-09	445
	Molecular Transport	9.18E-04 - 5.39E-09	307
EC vs CCC	Cellular Movement	5.27E-06 - 1.12E-26	606
	Cell Death and Survival	4.37E-06 - 9.65E-18	752
	Molecular Transport	1.93E-06 - 2.39E-16	514
	Carbohydrate Metabolism	3.73E-06 - 5.59E-13	268
	Cellular Development	5.41E-06 - 7.15E-12	723
EC vs MC	Drug Metabolism	6.88E-03 - 8.89E-13	32
	Lipid Metabolism	7.95E-03 - 1.97E-08	60
	Small Molecule Biochemistry	9.17E-03 - 1.97E-08	78
	Vitamin and Mineral Metabolism	7.66E-03 - 1.97E-08	36
	Energy Production	9.17E-03 - 2.04E-05	11
HGSC vs CCC	Cellular Movement	7.03E-07 - 1.82E-23	915
	Cell Death and Survival	6.72E-07 - 3.64E-20	1184
	Molecular Transport	9.68E-07 - 6.34E-19	752
	Cellular Development	8.55E-07 - 9.27E-15	971
	Cellular Growth and Proliferation	4.73E-07-9.27E-15	861
HGSC vs EC	Cellular Movement	2.85E-04 - 4.63E-17	316
	Cellular Function and Maintenance	2.10E-04 - 2.83E-10	359
	Molecular Transport	2.10E-04 - 5.21E-09	286
	Cell-To-Cell Signaling and Interaction	2.66E-04 - 1.85E-08	240
	Lipid Metabolism	1.94E-04 - 1.28E-07	174
HGSC vs MC	Carbohydrate Metabolism	1.32E-04 - 1.04E-09	172
	Post-Translational Modification	5.53E-04 - 4.79E-09	157
	Cellular Movement	1.69E-03 - 1.85E-08	485
	Cell Death and Survival	1.60E-03 - 2.62E-08	679
	Lipid Metabolism	1.70E-03 - 2.63E-08	253

Supplementary Table 5. Mutations found in the identified putative oncogenes and tumor suppressor genes of Table 1.

Gene symbol	Number of mutations	Mutation type	Mutation frequency
		**CCC case vs MC control**	
FAM20A	3	frameshift deletion, nonsynonymous SNV, synonymous SNV	1.05%
LAMB1	4	frameshift insertion, nonsynonymous SNV, synonymous SNV	1.05%
		**EC case vs CCC control**	
CLMN	2	frameshift insertion, nonsynonymous SNV	1.05%
		**HGSC case vs CCC control**	
CACNA1A	3	frameshift insertion, nonsynonymous SNV, synonymous SNV	1.05%
CACNB1	1	synonymous SNV	1.05%
CELF4	-	-	-
CLMN	2	frameshift insertion, nonsynonymous SNV	1.05%
COL14A1	5	frameshift insertion, synonymous SNV	1.05%
EBF4	4	frameshift deletion, nonsynonymous SNV, stopgain	1.05%
EHF	1	synonymous SNV	1.05%
HMGA2	-	-	-
IDH3B	2	nonsynonymous SNV, synonymous SNV	1.05%
KCNMB2	2	nonsynonymous SNV, stopgain	1.05-2.11%
LINC00578	-	-	-
MAPK4	1	synonymous SNV	1.05%
MEIS2	5	frameshift deletion, frameshift insertion, nonsynonymous SNV	
MTBP	1	frameshift insertion	9.47%
MYLK2	2	nonsynonymous SNV, synonymous SNV	1.05%
NRSN2	2	nonsynonymous SNV, stopgain	1.05%
PDYN	-	-	-
PPP1R1B	-	-	-
PROKR2	-	-	-
RASSF2	-	-	-
RPL22L1	-	-	-
TP63	3	frameshift deletion, nonsynonymous SNV	1.05%
ELP3	-	-	-
		**HGSC case vs EC control**	
AARD	2	frameshift deletion, synonymous SNV	1.05%
CCNE1	2	frameshift deletion, synonymous SNV	1.05%
CTCFL	3	frameshift deletion, nonsynonymous SNV, synonymous SNV	1.05%
LINC00578	-	-	-
LINC01532	-	-	-
RBM38	7	nonsynonymous SNV, synonymous SNV, stopgain	1.05%
RSPO4	-	-	-
UQCRFS1	1	nonsynonymous SNV	1.05%
URI1	-	-	-
PDE8B	5	frameshift deletion, frameshift insertion, nonsynonymous SNV	1.05%
		**HGSC case vs MC control**	
ANKS1B	2	nonsynonymous SNV	1.05%
COLEC10	1	nonsynonymous SNV	1.05%
EGFEM1P	-	-	-
KCNMB2-AS1	2	nonsynonymous SNV, stopgain	1.05-2.11%
LINC00578	-	-	-
MYEF2	1	frameshift deletion	1.05%
PDYN	-	-	-
RBFOX1	-	-	-
SNAP25	-	-	-
STON2	1	nonsynonymous SNV	1.05%
SULF1	-	-	-
TSHR	2	frameshift deletion, nonsynonymous SNV	1.05%
TGFBR2	6	frameshift deletion, frameshift insertion, nonsynonymous SNV,	1.05-2.11%

Supplementary Table 7. Number of patients analyzed in the different methods in view of histotypes.

	RNA-seq	DNA methylation	CNA
**CCC**	17	15	15
**EC**	17	16	16
**HGSC**	50	50	50
**MC**	11	10	10
**Total:**	95	91	91
